# Sleeplessness in Infants

**Published:** 1871-08

**Authors:** Eustace Smith


### PART III.

### Correspondence, Hygenic and Miscellaneous.

### SLEEPLESSNESS IN INFANTS.

BY DR. EUSTACE SMITH.

If the infant be very young, hunger is commonly the cause to which his restlessness is attributed by the mother; for the tendency of mothers is to refer all crying in their infants to that one cause. Occasionally they may be right. Infants nourished solely by the breast, and deriving their support from a scanty supply of watery milk, are almost constantly hungry. The amount of fluid they swallow is scarcely sufficient to satisfy their appetite even for the time; and, being rapidly digested, the meal is soon followed by renewed demands for nourishment. After a few days of such a diet, the weakly condition of the infant, induced by semi-starvation, draws attention to his state of health; but crying at night from hunger is an invariable forerunner of his loss of flesh.

By far the most common cause of restlessness at night is *injudicious feeding*, the child being stuffed with food, although not necessarily in itself injurious, is yet ill-adapted to the nourishment of the particular infant to whom it is given. It is a common practice among mothers—especially those of the poorer classes—to make up any deficiency in the amount of breastmilk by farinaceous food, long before the digestive power of the child is suited to such a diet. The stomach of an infant of about two months old is often filled with a mass of starchy matters, which the absence of saliva will not permit him to digest. This mass, fermenting in his bowels, is a source of continual discomfort until it is evacuated. Even when cows' milk is used as an addition to the breast-milk, it is very frequently ill-digested, although diluted with water.

*Cold feet* are a not unfrequent cause of wakefulness in infants. Delicate infants, in whom the circulation is languid, are very subject to coldness of the extremeties; and griping pains in the belly are common accompaniments of the same condition. In all cases of abdominal pain in infants the feet should be examined. When these are found to be cold, warming them by frictions with the hand, or by hot applications, usually causes the manifestations of pain to cease.

The feet in infants should be always carefully warmed before the children are put to bed; and should, in cold weather, be afterwards wrapped in flannel, or be covered with thick woollen socks.

In *hereditary syphilis*, infants are exceedingly fretful at night; and by their uncontrollable crying, are a source of great distress to the mother. This symptom is usually the first sign of the disease, preceding the snuffling and the other characteristic symptoms of the outbreak of the inherited taint. The crying is possibly excited by nocturnal pains in the bones, similar to those affecting the adult previous to the outbreak of the constitutional symptoms. On the appearance of the rash, the sleeplessness does not subside, but it soon disappears under the influence of specific treatment—a few doses of gray powder being sufficient to produce this result.

*Worms*, in older children, are well known to be a common cause of night terrors and restlessness; but even in infants, crying at night is sometimes found to be due to this cause. Amongst the poorer classes, where infants are allowed early to share in their parents' meals, it is not so very uncommon to find them suffering from the presence of oxyuris vermicularis. To give one instance out of many which have lately come under my notice: A child of nineteen months, well nourished, strong on his legs, who had walked from the age of ten months, had cut eighteen teeth, and could talk, the mother said, well, was brought for fits of violent screaming, which began about 8 p. m., and lasted the greater part of the night. From the condition of the tongue, worms were suspected, and a purgative of rhubarb and jalap brought away a large quantity of the small thread-worms. Afterwards, a careful regulation of the diet, and the administration of compound decoction of aloes, with a little iron, soon restored the alimentary canal to a healthy condition. The night screaming ceased from the very commencement of the treatment.

Besides the causes which have been enumerated, there are two others of not uncommon occurrence, and are frequently overlooked. One of these is the influence of *habit* upon the infant. Children who are too much petted and indulged, easily contract habits which are sources of great annoyance, not only to themselves, but also to those through whose uncalculating tenderness the habit has been acquired. Thus, in young children little attention should be paid to cries excited by other causes than actual suffering or discomfort. Cries from wilfulness or fretfulness should be entirely disregarded.

*Exhaustion of nerve-force*, the re-action following over-excitement of the nervous system, is another not uncommon cause of wakefulness at night in children. Children of three or four years old, after the excitement of a child's party, or a visit to some place of amusement, are often found to be troubled with sleeplessness; the child either finding a difficulty to compose himself to sleep, or waking up after a short slumber. The same thing is frequently seen in young infants who have been played with and over-excited immediately before being put to bed. The infant is uneasy and restless, starting frequently, and waking up with a fearful cry. This is not found with all infants, but is especially noticeable in those of delicate organization and great impressibility of the nervous system, and is, therefore, a frequent symptom of commencing rickets, where the irritability of the nervous system is very great.

Sleeplessness in infants is thus produced by many different causes, each of which will require a different method of treatment for its removal. To look upon such a condition as a distinct disease, removable by any so-called specific, is in the highest degree unphilosophical and unpractical. Opiates, and perhaps bromide of potassium, may be occasionally useful in quieting excessive irritability of the nervous system, and may be, therefore, of service in the treatment of sleeplessness arising from the last two causes which have been mentioned; but to employ either as a universal remedy in such cases, would be at least useless, even if it were not injurious. The screams of an infant suffering from an accumulation of undigested food—to take the commonest case—may certainly be quieted for the time by a narcotic; but so long as the cause remains, the screams will be renewed as soon at the soporic effect of the drug has had time to pass away. In such a case, bromide of potassium produce no effect whatever. In every case of sleeplessness in infants, the cause may be easily ascertained by careful investigation; and, when it is discovered, there is little difficulty about its removal.—*Medical and Surgical Reporter.*

*Carbolic Acid in Obstinate Leucorrhœa.*—Dr. Whitaker, of Cin. (*Cin. Lancet and Observer*), speaks highly of carbolic acid in the treatment of obstinate leucorrhœa. The application is made upon the cotton-wrapped sound. The instrument, so enveloped, is first introduced and the uterine surface cleansed; a new layer of cotton is substituted, medicated by immersion in a solution of proper strength, and applied to every portion of the uterine cavity.

